# Differences in survival between two cohorts of COPD patients living in Mexico City: a 20-year cohort comparison

**DOI:** 10.36416/1806-3756/e20250271

**Published:** 2026-08-13

**Authors:** Gustavo I Centeno-Saenz, Robinson Robles-Hernández, Alejandra Ramírez-Venegas, Sandra P Guinto-Ramirez, Rafael Hernández-Zenteno, Francisco López-Montiel, Abraham Tolentino-de la Mora, Justino Regalado, Rogelio Perez-Padilla

**Affiliations:** 1. Instituto Nacional de Enfermedades Respiratorias “Ismael Cosío Villegas” -INER - Ciudad de México, México.

**Keywords:** Pulmonary disease, chronic obstructive, Survival analysis, Prognosis, Cohort studies, Tobacco smoking

## Abstract

**Objective:**

To compare the survival rates for two cohorts of COPD patients living in Mexico City and studied 20 years apart at the same referral center, with demographic characteristics, lung function, hypoxemia, type of exposure associated with COPD, and socioeconomic status being analyzed.

**Methods:**

Two cohorts of COPD patients were studied and followed at a single referral center for respiratory diseases in Mexico City, at an altitude of 2,240 m above sea level. The first cohort was studied between 1996 and 2003, and the second was studied between 2016 and 2023. Participants were followed for seven years, and a multivariate Cox model was used in order to analyze survival.

**Results:**

The first cohort included 434 patients, and the second comprised 416 patients, with a total of 850 patients being analyzed. Of those, 68% had COPD from tobacco smoking and 32% had COPD from biomass smoke exposure. In the multivariate Cox model adjusted for age, sex, smoking status, exposure to biomass smoke, BMI < 21 kg/m^2^, hypoxemia, six-minute walk distance, and lung function as assessed by spirometry, the 2016-2023 cohort showed a significant reduction in the seven-year mortality rate (hazard ratio = 0.59; 95% CI, 0.44-0.81; p = 0.001) when compared with the 1996-2003 cohort.

**Conclusions:**

Survival of COPD patients has improved over the past 20 years after accounting for several mortality predictors.

## INTRODUCTION

COPD persists as the third leading cause of death, with an estimated world prevalence of 10.3%.^1^ In Mexico, COPD was the leading cause of death from chronic respiratory diseases in the 1990-2021 period. 

Since the 1990s, the age-standardized and crude mortality rates for COPD have shown a downward trend worldwide,^2^ particularly among men. However, the number of deaths continues to rise.^3^ This improvement in global survival may be linked to the availability of smoking cessation programs and widespread implementation of oxygen therapy for hypoxemic patients,^4^ smoking cessation and oxygen therapy being the initial interventions that have been shown to improve survival in COPD.^4^ Other interventions have also proven beneficial, such as nocturnal ventilation in hypercapnic patients,^5^ pulmonary rehabilitation,^6^ and recent developments in inhaled therapy.^7^ Treatment and prevention of exacerbations are expected to reduce the numbers of complications and deaths.^8^ Therefore, there was a general trend toward increased life expectancy in most countries, at least before the COVID-19 pandemic. 

In Latin America, survival of patients with COPD has also improved according to the 2021 Global Burden of Disease Study^9^; however, underdiagnosis persists,^10^ along with sex disparity and unfavorable socioeconomic conditions, likely leading to a worse prognosis, particularly in women with biomass smoke-related COPD in comparison with women with tobacco smoke-related COPD.^11^ ^,^ ^12^ In addition, increased altitude of residence may increase COPD mortality.^13^ Patients with COPD may experience a decrease in exercise capacity, an increase in dyspnea, and more severe hypoxemia as a result of decreased inspired oxygen pressure.^14^ In residents of Mexico City and the Valley of Mexico, one of the interventions that would be expected to impact survival is the use of long-term oxygen therapy, access to which has gradually increased. According to a study of COPD prevalence in five Latin American cities, the prevalence of an SpO_2_ of ≤ 88% was estimated at 6% in individuals over 40 years of age residing in the Mexico City metropolitan area.^15^

We aimed to compare the survival rates for two cohorts of COPD patients living in Mexico City and studied 20 years apart at the same referral center. We investigated whether there was an improvement in survival at a single referral center for respiratory diseases and subsequently explored factors contributing to the improvement. 

## METHODS

We conducted a survival analysis of two cohorts of patients with COPD caused by tobacco smoking or biomass exposure. They were studied 20 years apart, being each followed by seven years at the Smoking and COPD Clinic of the *Instituto Nacional de Enfermedades Respiratorias “Ismael Cosío Villegas*,*”* located in Mexico City. The *Instituto Nacional de Enfermedades Respiratorias “Ismael Cosío Villegas”* is a referral center and research institute for respiratory diseases located at 2,240 m above sea level and mostly caring for uninsured patients. Patient data were collected and coded for anonymity. The original cohorts and the present study were approved by the local research ethics committee (Protocol nos. C06-13 and C08-05), and all patients provided written informed consent. 

### Study population and materials

We included all cases assessed during the study period and followed from the recruitment date. The status of alive or dead was corroborated at seven years: if the latest medical notes were older than six months or if the medical record was unavailable at the clinic, phone calls were made to the patient or relatives, and, finally, if necessary, we searched the electronic death certificate database of our country to confirm a deceased status. We did not analyze the cause of death when available in a death certificate, because we considered that it could be imprecise. Loss of follow-up was defined as informative censoring. 

Chronic respiratory symptoms were reported by all participants, and the spirometric criteria for COPD diagnosis were in accordance with the American Thoracic Society/European Respiratory Society (ATS/ERS) standards (i.e., a post-bronchodilator FEV_1_/FVC ratio of < 0.70).^16^ Exposure was classified as tobacco smoke-related COPD if a cumulative smoking history > 10 pack-years was reported or as biomass smoke-related COPD if a cumulative exposure > 100 hour-years (years cooking in an open fire times the average number of hours a day spent cooking) was reported.^17^ We described previous exacerbations only for the 2016-2023 cohort because during the earlier cohort period (1996-2003) we lacked systematic recording of COPD exacerbations; a formal consensus-oriented definition was later proposed by Rodríguez-Roisin, underpinning contemporary exacerbation ascertainment.^18^

Data from the 1996-2003 cohort were previously reported by Ramirez et al.^12^ All patients underwent physical examination and the six-minute walk test following the protocol proposed by McGavin et al.,^19^ and the six-minute walk distance (6MWD) was expressed as a percentage of predicted values calculated by the reference equations proposed by Enright & Sherrill.^20^ Post-bronchodilator spirometry was performed with a dry rolling-seal volume spirometer (SensorMedics, Yorba Linda, CA, USA) following ATS-recommended procedures^15^ and Third National Health and Nutrition Examination Survey reference values for Mexican-Americans.^21^

Spirometry for the 2016-2023 cohort was performed with an EasyOne Pro spirometer (ndd Medical Technologies, Inc., Zurich, Switzerland) in accordance with the ATS/ERS guidelines.^22^ The six-minute walk test was conducted in accordance with a protocol validated by the ERS/ATS, with the 6MWD being reported in meters and as a percentage of the predicted value.^23^

A qualified social worker carried out a routine socioeconomic status (SES) assessment, considering the family’s monthly income adjusted and updated for inflation, based on multiples of the Mexican national minimum wage. This income, stratified into deciles, corresponds to the total sum of the earnings of the economically active household members. However, the dependents on the total family income were also considered. This official SES classification is utilized by public hospitals and health centers. SES ranged from 0 to 6, and the level determined the health care cost for the patient. SES was divided later into three categories: low income (0-2), middle income (3-4), and high income (5-6). 

### Statistical analysis

Survival in the two cohorts was compared by Cox proportional hazards models adjusted for known and possible predictors of survival: age; sex; type of primary exposure in COPD (biomass smoke or tobacco smoking); severity of obstruction by FEV_1_ categories according to the GOLD^24^; resting SpO_2_ < 90%; 6MWD (in % of predicted); and persistent tobacco smoking, as applicable. Survival function was estimated for fixed periods at three, five, and seven years in both cohorts, along with death incidence rates, by sex and exposure to tobacco smoke or biomass smoke, for a clearer comparison between the two cohorts. 

We also fitted a multivariable Poisson regression model to estimate mortality incidence rate ratios (IRRs) with the same covariates. In addition, we used logistic regression to explore the influence of previous exacerbations and the relationship between mortality-related factors. 

Initial univariate analysis assessed factors influencing survival rates in addition to known or previously described risk factors for death. Differences between survivors and non-survivors in lung function variables and other relevant characteristics were evaluated using independent-sample t-tests and the chi-square test for categorical variables. Adjusted hazard ratios (HRs) and 95% confidence intervals were calculated in the multivariate model, and their suitability was assessed by residue analysis. Statistical analysis was performed with Stata, version 13.1 (StataCorp LLC, College Station, TX, USA), a significance level of 5% being considered. 

## RESULTS

Eight hundred and fifty Mexican patients with COPD were studied, with both cohorts predominantly having tobacco smoke-related COPD over biomass smoke-related COPD (68% vs 32%). The 1996-2003 cohort included 434 patients, of whom 258 were men and 176 were women. Of those, 311 were part of the tobacco smoke-related COPD group, with a majority being men (76.5%). The remaining 123 patients, most of whom were women (83.7%), belonged to the biomass smoke-related COPD group.

The 2016-2023 cohort consisted of 416 patients, including 198 men and 218 women. Within this group, 272 patients were classified as having tobacco smoke-related COPD, with men predominating (69.1%), whereas 144 patients were classified as having biomass smoke-related COPD, with women predominating (93.1%; Table 1). 

**Table 1:** Demographic and clinical variables, by cohort of COPD patients.^a^

	1996-2003 (n = 434)	2016-2023 (n = 416)	p
Age, years	68.2 ± 9.4	72.5 ± 9.8	< 0.001
Male	59.5 (258)	47.6 (198)	0.001
Current tobacco smoking	8.5 (37)	8.9 (37)	0.823
Smoking history, pack-years*	48 [11-198]	40 [10-123]	< 0.001
Biomass smoke exposure, hour-years**	228 [102-720]	300 [102-780]	0.002
Socioeconomic status			
Low	53.9 (195)	67.7 (256)	
Middle	38.4 (139)	31.8 (120)	< 0.001
High	7.7 (28)	0.5 (2)	
Clinical characteristics			
BMI, kg/m^2^	25.3 ± 4·6	26.2 ± 5.7	0.024
mMRC dyspnea scale score	2.1 ± 1·4	1.9 ± 1.2	0.054
GOLD stage			
1	16.1 (70)	22.3 (93)	0.010
2	38.5 (167)	42.1 (175)	
3	28.3 (123)	24.3 (101)	
4	17.1 (74)	11.3 (47)	
6MWD, m	402 ± 187.3	361 ± 112.2	< 0.001
6MWD, % predicted	86.5 ± 39.7	84.4 ± 25.1	0.359
FEV_1_, L	1.15 ± 0.53	1.21 ± 0.58	0.137
FEV_1_, % predicted	53.8 ± 25.4	60.9 ± 29.7	< 0.001
FVC, L	2.29 ± 0.84	2.27 ± 0.9	0.668
FVC, % predicted	72.3 ± 20.9	83.4 ± 28.9	< 0.001
FEV_1_/FVC	0.50 ± 0.13	0.54 ± 0.15	< 0.001
SpO_2_	88.1 ± 5.3	88.2 ± 5.2	0.778
Moderate exacerbation	-	36.5 (148)	-
Severe exacerbation	-	6.2 (52)	-
Frequent exacerbation	-	7.8 (66)	-

mMRC: modified Medical Research Council; and 6MWD: six-minute walk distance. ^a^Data presented as % (n), mean ± SD, or median [min-max]. *1996-2003 cohort = 311 patients; 2016-2023 cohort = 272 patients. **1996-2003 cohort = 123 patients; 2016-2023 cohort = 144 patients.

The 2016-2023 cohort had an older mean age, lower cumulative smoking in pack-years, higher BMI, better FVC in % of the predicted value, better FEV_1_ in % of the predicted value, and lower 6MWD (Table 1). No significant differences were observed between the two cohorts regarding mean SpO_2_ or dyspnea as measured by the modified Medical Research Council scale. The proportion of patients with low SES increased in the second cohort. In multivariate models, active smoking consistently increased mortality (HR = 3.17; 95% CI, 2.18-4.60; p < 0.001), as did an SpO_2_ of < 90% (HR = 2.32; 95% CI, 1.58-3.39; p < 0.001) in both cohorts. 

**Figure 1:**
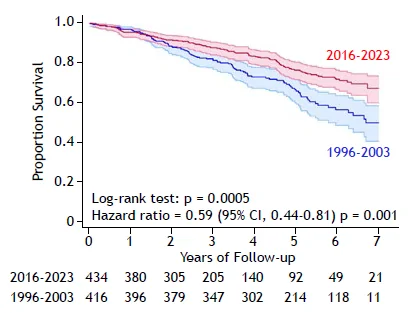
Comparison of Cox models between cohorts, adjusted for age, sex-exposure interaction, current smoking, FEV_1_ < 30% of the predicted value, six-minute walk distance (in % of the predicted value), and SpO_2_ < 90%.

**Figure 2:**
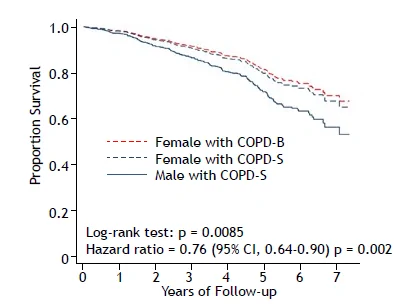
Comparison of survival between patients with biomass smoke-related COPD (B-COPD) and those with tobacco smoke-related COPD (S-COPD), by sex and adjusted for age, current smoking, socioeconomic status, FEV_1_ < 30% of the predicted value, six-minute walk distance (in % of the predicted value), and SpO_2_ < 90%.

Crude seven-year mortality was significantly lower in the 2016-2023 cohort than in the 1996-2003 cohort (HR = 0.67; 95% CI, 0.61-0.73; Table 2). After adjustment, mortality in the 2016-2023 cohort remained markedly lower-by more than 40%-when compared with that in the 1996-2003 cohort (adjusted HR = 0.59; 95% CI, 0.44-0.81; p = 0.001; Table 3 and Figure 1). Older age, male sex, hypoxemia (SpO_2_ < 90%), active smoking, a BMI of < 21 kg/m^2^, a lower 6MWD, and an FEV_1_ of < 30% of the predicted value were independent factors associated with mortality (Table 3). In adjusted multivariate models, women had a lower mortality than did men with tobacco smoke-related COPD (Figure 2 and Table 3). 

**Table 2:** Comparison of survival rates between the two cohorts of COPD patients.

Time	Fail	Survivor function (95% CI)	Fail	Survivor function (95% CI)
Cohort	1996-2003		2016-2023	
1 year	54	0.96 (0.94-0.98)	20	0.95 (0.93-0.97)
5 years	102	0.67 (0.60-0.72)	88	0.77 (0.72-0.81)
7 years	130	0.51 (0.42-0.59)	107	0.67 (0.61-0.73)

Note: The incidence rate of death is calculated on the basis of the number of years and deaths per 1,000 exposed individuals.

**Table 3:** Mortality predictors and survival in the two cohorts of COPD patients.

	Univariate analysis		Multivariate analysis	
	HR (95% CI)	p	HR (95% CI)	p
Age, years	1.03 (1.01-1.05)	< 0.001	1.04 (1.02-1.06)	< 0.001
Men/women	1.54 (1.17-2.02)	0.002	1.41 (0.97-2.06)	0.074
Biomass smoke-related COPD	0.72 (0.53-0.98)	0.035	0.95 (0.61-1.46)	0.800
FEV_1_ > 80% of the predicted value	Ref	-	-	-
FEV_1_ = 51-80% of the predicted value	0.93 (0.62-1.41)	0.748	-	-
FEV_1_ = 31-50% of the predicted value	1.38 (0.90-2.11)	0.136	-	-
FEV_1_ < 30% of the predicted value	2.84 (1.84-4.39)	< 0.001	1.77 (1.21-2.61)	0.004
SpO_2_ < 90%	2.94 (2.11-4.09)	< 0.001	2.32 (1.58-3.39)	< 0.001
Current smoking	4.34 (3.14-6.01)	< 0.001	3.17 (2.18-4.60)	< 0.001
BMI < 21 kg/m^2^	2.48 (1.82-3.37)	< 0.001	1.60 (1.13-2.27)	0.008
6MWD, % predicted	0.98 (0.97-0.99)	< 0.001	0.99 (0.98-0.99)	< 0.001
Low income	1.03 (0.77-1.38)	0.839	-	-
Middle income	Ref	-	-	-
High income	0.76 (0.31-1.89)	0.562	-	-
2016-2023 cohort vs. 1996-2003 cohort	0.62 (0.47-0.81)	0.001	0.59 (0.44-0.81)	0.001

Ref: reference; and 6MWD: six-minute walk distance.

The 2016-2023 cohort had lower mortality IRRs than did the 1996-2003 cohort (IRR = 0.51; 95% CI, 0.37-0.72; p < 0.001). The mortality IRR predictors in Poisson models were as follows: older age (IRR = 1.04; 95% CI, 1.02-1.05; p < 0.001); GOLD 4 COPD (IRR = 1.74; 95% CI, 1.19-2.52; p = 0.004); hypoxemia (IRR = 2.24; 95% CI, 1.55-3.24; p < 0.001); current smoking (IRR = 3.53; 95% CI, 2.52-4.94; p < 0.001); and a BMI of < 21 kg/m^2^ (IRR = 1.53; 95% CI, 1.15-2.16; p = 0.005). However, the 6MWD in % of the predicted value was protective (IRR = 0.991; 95% CI, 0.986-0.996; p = 0.001). The low-income group showed higher mortality (IRR = 1.54; 95% CI, 1.13-2.10; p = 0.006; Table S1). 

In the 2016-2023 cohort, the model for total exacerbations showed a significant association with a modified Medical Research Council scale score ≥ 2 (OR = 3.07; 95% CI, 1.91-4.96; p < 0.001) and an inverse association with younger age (OR = 0.96; p = 0.004). In the adjusted multivariate models, biomass smoke exposure showed no further independent association with survival or death, and categorical SES was not significant (Table S2). 

## DISCUSSION

In the present study, we found a significant improvement in survival among patients with COPD treated at a respiratory referral center in Mexico City over 20 years, even after accounting for major demographic, clinical, and socioeconomic predictors of mortality. 

The improvement in life expectancy observed in the cohorts significantly exceeds the increase in life expectancy of the Mexican population of similar age. Life expectancy at birth between 1996 and 2016 rose by 2.4 years from 72.0 to 74.4 years.^25^ However, the health-adjusted life expectancy at the mean age of the 1996-2003 cohort (68 years, utilizing estimates from 65-69 years) was 12.16 years (95% CI, 10.9-13.2), whereas, in the 2016-2023 cohort, it was 13.15 years (95% CI, 11.8-14.3), an expected increase of only one-year average survival in a general population cohort of Mexicans of a similar age to that of the patients.^26^

The improvement in survival observed in our patients takes into account known predictors, such as age, level of lung function as assess by FEV_1_ in % of the predicted value, and oxygenation (SpO_2_), especially relevant at moderate and high altitudes, with a known adverse impact on survival in patients with COPD,^27^ ^,^ ^28^ including our patients, followed in Mexico City.^12^ Persistence of active smoking despite the availability of effective treatments was also taken into account,^29^ as was the presence of a low BMI, associated with a poor prognosis in other studies.^30^

In both cohorts of COPD patients in the present study, smokers had more severely impaired lung function (and, consequently, an expected worse prognosis) when compared with those exposed to biomass smoke, although there was no substantial difference in the level of oxygenation, air trapping, or other clinical indicators such as limitation of activities and quality of life.^31^ ^,^ ^32^ However, after adjusting for the predictor variables, we found that worse survival persisted in men who developed COPD because of smoking when compared with women who developed COPD because of exposure to biomass smoke while cooking, corroborating the previously published results for the first cohort of patients.^12^

The importance of quitting smoking in patients with COPD stands out, being the strongest predictor of mortality in our study (HR = 3.17). Stopping tobacco smoking tends to be overlooked amid the avalanche of inhaled medications and pharmacological interventions. Patients with COPD require intensive intervention to stop smoking as a vital and cost-effective measure.^33^

Another relevant factor was an SpO_2_ of < 90% (HR = 2.32), which is especially important in countries where hypoxemia associated with moderate and high altitudes is prevalent, leading to increased mortality and complications.^27^ ^,^ ^28^

Changes in mortality between two U.S. COPD cohorts recruited in the 1971-1975 and 1988-1994 periods were previously described.^34^ They found that the age-adjusted mortality rate decreased by 15.8% for those with moderate or severe COPD, although this decrease was not significant. The reduction was more pronounced in those with mild COPD and those with normal lung function. One group of authors observed a 48% reduction in five-year mortality among patients with severe/very severe COPD in the 2000s when compared with those in the 1990s, attributing this improvement to long-acting bronchodilators.^35^ One study highlighted that COPD is a strong predictor of mortality, independent of cardiovascular and metabolic comorbidities.^36^

A third factor to consider was the type of exposure and sex, as we found a higher mortality rate in men with tobacco smoke-related COPD than in women with tobacco smoke-related COPD or biomass smoke-related COPD, which other authors have also discussed.^37^ ^,^ ^38^

In our study, older age, very advanced disease (GOLD 4 COPD), hypoxemia, and reduced functional reserve were consistently associated with higher mortality. The protective effect of the 6MWD aligns with the established role of exercise capacity and functional performance as key determinants of prognosis. Likewise, the increased risk associated with a BMI of < 21 kg/m^2^ is consistent with the poor prognosis phenotype related to low body mass, muscle wasting, and the systemic component of COPD. 

In contrast, neither biomass exposure nor its interaction with the studied cohort reached statistical significance. This suggests that the effect of biomass smoke-related COPD on mortality was explained in our study by disease severity and functional measures. Alternatively, the study may have limited power to detect effect modification by exposure type. 

Although we reported the experience of a single referral center, this considerably improves comparability between the two cohorts of COPD patients. Another limitation of the present study is the lack of information on severe exacerbations in the first cohort, which precluded its use in the survival analyses. On the other hand, several of the predictors of survival are also predictors of exacerbations, including, for example, obstruction severity, dyspnea, and current smoking.^39^ ^,^ ^40^

The absence of detailed treatment data reflects the historical context of the earlier cohort, when COPD management was limited mainly to short-acting bronchodilators, whereas current survival gains likely reflect advances in long-acting inhaled therapies and overall disease management when stable and during exacerbations. In addition, the cause of death of our patients could not be accurately determined, nor could it be adjusted by cardiovascular, metabolic, or other comorbidities, especially relevant during the SARS-CoV-2 pandemic. 
